# Social touch: intertwining with embodied others

**DOI:** 10.3389/fpsyg.2023.1171062

**Published:** 2023-06-15

**Authors:** Wei Chen, Huanhuan Ma, Da Dong

**Affiliations:** ^1^Center for Brain, Mind and Education, Shaoxing University, Shaoxing, China; ^2^Department of Psychology, Shaoxing University, Shaoxing, China; ^3^Interdisciplinary Center for Philosophy and Cognitive Sciences, Renmin University of China, Beijing, China

**Keywords:** social touch, social interaction, multimodal video recording, embodied intersubjectivity, affective touch

In a passage at the end of *De anima* 2.11, Aristotle discusses how the constitution of the organ of touch affects its sensitivity, in a way that has implications for the kind of changes involved. The organ of touch is unique among the senses. In the other senses, the material is *neutral* with respect to the range in question: the eye jelly, for example, is colorless, the air in the ear silent. Touch, in contrast, inevitably possesses some of the qualities along its own range.

(Caston, 2005, p. 285)

In recent years, researchers have been captivated by one kind of sensory modality closely related to social interaction—touch. It is a basic sense developed at birth that plays an important role in inducing and regulating human cognition and emotion (Montagu, 1971; Gallace and Girondini, 2022). In the embodied-social behavioral settings, touch is a particular sensuous practice in contrast to the use of other sensations. Although touch is widely discussed across different disciplines and there is a growing literature on the subject, however, these different approaches are hardly combined; and it is not quite clear how touch is systematically used and deployed in social interactions. Particularly, in this ongoing pandemic, this important and fundamental sensation possibly may have been overlooked because of the forced social isolation (cf. Bohic and Abraira, 2022). Therefore, more systematic considerations on the diverse and broader settings of social interactions need to be presented.

Given the significance of touch and the shortcomings of current research on touch as mentioned above, the book, *Touch in Social Interaction: Touch, Language, and Body*, edited by Asta Cekaite and Lorenza Mondada, attends to pave the way “for conceptual reflections about the consequences of fully integrating the sense of touch in the study of the organization of social interaction” (Cekaite and Mondada, 2021, p. 12). It establishes a comprehensive foundation for current perspectives of touch in social interaction. In the framework proposed in this book, touch is considered as a distributed and collectively experienced capacity rather than a separated sensation (or merely a tool for personal enjoyment) (cf. Schirmer et al., 2022). The book consists of fourteen chapters, including an introduction (Chapter 1), a main body (Chapters 2–13), and a conclusion (Chapter 14). According to the different objects and types of touch which have been classified, here we attempt to divide the book into two parts: interpersonal touch (Part I) and material touch (Part II). Part I includes Chapters 2 to 10, which deals with touch between interpersonal contacts. This section focuses on the theme of why something other than physical contacts plays more of a role in the occurrences of touch. Part II (Chapters 11–13) aims to explore “how humans access, perceive and form the representation of the environment through tactile experiences” (p. 4), which involves material touch in different contexts and expands our scope on how we interact with the proximal, material surroundings.

Serving as the general overview of the book, Chapter 1 shows the significance of touch and its social role. It presents the latest progress of research on touch from a multidisciplinary perspective, and offers a multimodal interactive analysis method on studying touch in social interaction.

Touch is closely associated with emotion (Ravaja et al., 2017), which can be achieved interactively during a hug. Marjorie Goodwin in Chapter 2 focuses on the emotional dimension of touch and explores the trajectories of hugs under both the dyadic and multiparty participation frameworks (cf. Packheiser et al., 2021). Not only can these two different participation frameworks work together, but also, they can easily shift between each other. This chapter demonstrates that the multimodal features of hugs, whether in terms of vocal quality, intertwining of bodies and facial expression, vary in the context of families and parties among friends. The next chapter (Chapter 3) continues to explore this affective dimension of touch, presenting parents and children kissing each other in different ways during family photography sessions in collaboration with a professional photographer. It reveals the organization and accountability of kissing in a visible and public way through photographing and witnessing (by others externally). Kissing is not only an intimate act, but also a socially institutional act. This chapter illustrates that kissing and photographing are organized sequentially; they are intertwined and coordinated with each other. Different touch actions can address different affective goals, thus it is possible to address the needs of oneself or the others in “a context- and relationship-specific manner” (Schirmer et al., 2021). Chapter 4 pays attention to the interactive practices between adults and children. It explores the adults' responses to the pain-caused distress of children; interactive practices include the mixing of physical touch—e.g., skin-to-skin contact, sustained embrace and other multisensory features. Touch as a mediator between the caregivers and children has pain-relieving and mood-regulating benefits. More importantly, it can convey affection and connection within intimate dyads. In Chapter 5, the authors quest for touch practices between adults and children in educational settings. They explain how preschool teachers use control touch and other multimodal resources to mediate the peer conflicts among young children in Sweden and Japan. Although there are noteworthy cross-cultural differences between these two distinct societies, we deem that the purpose of control touch is to guide children to engage in appropriate social behaviors and encourage them to become morally decent and socially active persons.

Chapters 2–5 show touch in naturally affectionate and controlling behaviors, followed by Chapters 6–7 that turn the attention in the dimension of disciplined physical activities. In Chapter 6, Leelo Keevallik examines the basic traditional dance hold of the Lindy Hop training and focuses on the pause owing to an upcoming dance projection. The author shows how students in a partner dance class treat the mutual touch as legitimate in the period before they practice their dance moves through an instance, in order to avoid discomfort of the dance hold when it is situationally inappropriate. It suggests that the use of touch in certain situations is monitored and guided by a specific sequence of interactions. Another instance is aikido, Augustin Lefebvre in Chapter 7 focuses on “touching-whole-body-moments” interaction, where aikido practitioners rely on physical contact as their primary resource to coordinate movement. “As soon as the bodies make contact, practitioners generate a shared whole-body-movement through two symmetric actions: to touch and to be touched.” (p. 151) In this case, coordination as an interactive phenomenon, occurs in a form in which sequentiality and simultaneity coexist and are interconnected.

Touch is not exclusive to humans, is also widely applicable to interactions between humans and animals, and is actually a crucial modality in human-animal communication. Chapter 8 extends the perspective of touch to the interactions between species, investigating how the sequences of petting by humans and dogs is completed mutually in training and in domestic settings. This chapter explains how the touch stemming from interspecies, especially stroking and petting, constitutes a socially important and fundamental medium in domestication. Besides, touch is a common and crucial resource in the therapeutic context, and it is often used by speech and language therapists for the treatment of post-stroke aphasia patients who have lost some or most of their linguistic and communicative abilities (Chapter 9). In this chapter, apart from three different modes of touch in the therapeutic practice, we can also see how and when they are used by therapists, as well as their impact on patient behaviors. In terms of medical settings, in Chapter 10, Aug Nishizaka reveals in Japanese midwifery practices, how pregnant women are guided to feel and identify the target object (the body part of the fetus) in a particular abdominal position. Notably, the author argues that further efforts should be paid to what the participants perceive in the interaction, rather than just the sequential context in which they perceive things. For example, the guidance of one's hands should be distinguished from the instruction on how to move the hands.

Touch is not only a way for us to interact with other persons, but also a channel to perceive our surroundings and its materiality. Touch is also the key to the success of the surgical procedures. For instance, in Chapter 11, Christian Heath and Paul Luff reveal how medical tools are grasped, felt, and used in a timely and relevant way between scrub nurses and surgeons; and they explore participants can sense and feel how and when these objects will be held by the recipients. This chapter is particularly concerned with the exchange of tools and implements, thereby uncovering the characteristics of tactile and sensory activities when people manipulate objects together. Scrub nurses and surgeons perceive and predict each other's intentions and trajectories of hands through the manipulation, grasping and handling of tools in the interaction generated by physical behaviors, for the aim of collaboratively completing the surgical procedures. Touch is also prevalent in geological realm. Charles Goodwin and Michael Smith in the following chapter reveal how geologists recognize, classify and acquire knowledge of rocks. This is done by instructing perception through touch in the practice of training geologists in the Yellowstone area. It can be seen that this geological practice is based on the geologists' tactile experiences of materials, which further forms a clear recognition of external objects (gradually stabilizing during the course of interactions). Chapter 13 reveals another form of professional touch that occurs in food practices. The author, Lorenza Mondada examines the seller's touch and the customer's touch when touching cheese. From their distinct practices, we can see that, touch, along with other multiple sensory accesses, is finely coordinated in ongoing collective actions, which is essential to ensure the intersubjectivity of shared sensorial experiences, evaluation of product quality and subsequent purchase decisions.

Finally, as an epilog, Chapter 14 addresses the ambivalences of touch through lovers' touch, and potential influences of violent touch, and further discusses several background literatures that are presented in Chapter 1.

To sum up, the chapters in this volume are of great methodological and conceptual value to the study of touch. First, methodologically, in contrast to the experimental studies, traditional interviewing and field notes used in many other studies on touch, this book adopts a comprehensive analytical perspective that combines multimodal interaction analysis with the method of video recording. The new approach emphasizes the systematic analysis of various multimodal resources that underlie social actions in the context. As for video recording, it provides essential materials and resources for analysis. From our perspective, similar to the traditional interviewing, video recording method needs to be carried out directly around the others, and aims to obtain the required data from the embodied others^1^. Yet the new multimodal method is superior to the traditional method in several aspects. For the convenience of comparison, three differences on multimodal video recording and the traditional interviewing are offered in Table 1. This new video method could be seen as an audio-visual representation of touch, and a way reconstructs the participant's perspective of “witnessing the event—revealing the social intelligibility of what happened” (p. 18). We reckon that this method has several advantages: for example, it takes place in the real-world situation and thus is not restricted by the situated locality; it facilitates the recording of inconspicuous cases that may provide detailed qualitative research analysis; and it is helpful to investigate the precise time and trajectory of the contact at the moment it appears. We suppose that with these advantages, this method can provide insights into complex situated activities, the temporality of movement, and analyzable multimodal resources.

**Table 1:** Three issues on which multimodal video recording and traditional interviewing differ.

**Issue**	**Multimodal video recording**	**Traditional interviewing**
Whether it is limited by the ability of the researcher	No	Yes; and it requires higher ability of the researcher
The nature of the data gathered	Objective	Subjective
In what context it is used	In a wide range of social interactions	Usually in the study of personality and individuality

Second, conceptually, it is clear that touch, as a peculiar mode of sensation associated with social interaction, is radically different from the other modalities. Tactile contact is more than a communicative behavior designed to express, communicate, or transmit social meanings. Further, the book conceptualizes touch as “a sociocultural phenomenon deeply rooted in social interaction” (p. 1) resorting to detailed studies of touch moments and processes of joint action in social contexts. In reading this book, the readers can have a relatively in-depth and comprehensive understanding of how touch is realized in the context of social interaction through multimodal resources.

In conclusion, we hold that the book provides different aspects of empirical cases from touch practices across different cultural contexts, which will help us understand this significant yet underexplored realm. It reveals how different practices and aspects of touch are systematically combined with multimodal features to generate meaning and comprehensibility of behaviors in social interactions.

Nevertheless, we thought it would be more comprehensive if the book had included more representative countries from Asia, Europe, Africa, etc.; because we know that touch varies considerably in different cultural contexts. In addition, we suspect multimodal video recording is to some degree cumbersome and requires more time and effort to improve. Furthermore, this collection of essays seems to have omitted special but very valuable cases, such as the cases of the visually impaired. Brock et al. (2015) identified mobility and orientation as one of the biggest challenges faced by visually impaired people. That is to say, for such people, visual barriers seem to prevent them from accessing tactile information, which in turn leads to interpersonal interaction barriers to some extent. On the basis that the rapid development of smart devices such as mobile phones, touch-based interfaces can be a huge challenge for most visually impaired people (Kane et al., 2008), as some gestures such as text editing require complex finger movements or the coordination of several fingers to complete (Buzzi et al., 2017). Although studies have shown that virtual reality can transform information and events excluded from impaired senses into those perceived by unimpaired senses, thus providing a safe and unrestricted environment for people with sensory impairments to experience things that cannot happen in real life (cf. Lorenzo et al., 2016; Potkonjak et al., 2016; Papanastasiou et al., 2018); based on the definition of “cyberspace” by Gibson (1984) and others, it is reasonable to insist that touch in virtual reality and in real interaction have significant differences and different effects, which is evident in this pandemic. Additionally, we are rather curious about the fact that we can interact virtually with each other through the touch of a digital screen, in the form of emoji, text and voice, and in which sense such an approach could be called multimodality. If this is the case, then what is the core differences between it and real interactive touch?

Notwithstanding, since the increasing interest in the research of touch, we maintain that this book is a timely and critical contribution to this immature subject, especially for those working in the fields of communicative analysis, multimodality, and other related realms.

## Author contributions

All authors listed have made a substantial, direct, and intellectual contribution to the work and approved it for publication.

## Footnotes

^1^The following remarks from Merleau-Ponty (1962) offers a classical explanation on the intersubjectively embodied (or, to use Merelau-Ponty's own word, “intercorporeal”) significance of touch: “The reason why I have evidence of the other man's being-there when I shake his hand is that his hand is substituted for my left hand, and my body annexes the body of another person in that ‘sort of reflection' it is paradoxically the seat of. My two hands ‘coexist' or are ‘compresent' because they are one single body's hands. … he and I are like organs of one single intercorporeality” (p. 168).
